# Analyses of hypoxia-related risk factors and clinical relevance in breast cancer

**DOI:** 10.3389/fonc.2024.1350426

**Published:** 2024-03-04

**Authors:** Yan Li, Haiyang Yu, Xinghua Han, Yueyin Pan

**Affiliations:** ^1^ Department of Clinical Oncology, The First Affiliated Hospital of University of Science and Technology of China (USTC), Division of Life Sciences and Medicine, University of Science and Technology of China, Hefei, Anhui, China; ^2^ Division of Life Sciences and Medicine, University of Science and Technology of China, Hefei, Anhui, China

**Keywords:** breast cancer, hypoxia, risk model, biomarkers, tumor microenvironment

## Abstract

**Introduction:**

Hypoxia plays an important role in the heterogeneity, relapse, metastasis, and drug resistance of breast cancer. In this study, we explored the hypoxia-related biological signatures in different subtypes of breast cancer and identified the key prognostic factors by bioinformatics methods.

**Methods:**

Based on The Cancer Genome Atlas (TCGA) Breast Cancer datasets, we divided the samples into immune-activated/suppressed populations by single-sample gene set enrichment analysis (ssGSEA) and then used hierarchical clustering to further identify hypoxic/non-hypoxic populations from the immune-suppressed samples. A hypoxia related risk model of breast cancer was constructed.

**Results:**

Nuclear factor interleukin-3 regulated (NFIL3), serpin family E member 1 (SERPINE1), FOS, biglycan (BGN), epidermal growth factor receptor (EGFR), and sushi-repeat-containing protein, X-linked (SRPX) were identified as key hypoxia-related genes. Margin status, American Joint Committee on Cancer (AJCC) stage, hypoxia status, estrogen receptor/progesterone receptor (ER/PR) status, NFIL3, SERPINE1, EGFR, and risk score were identified as independent prognostic indicators for breast cancer patients. The 3- and 5-year survival curves of the model and immunohistochemical staining on the breast cancer microarray verified the statistical significance and feasibility of our model. Among the different molecular types of breast cancer, ER/PR+ and HER2+ patients might have higher hypoxia-related risk scores. ER/PR-negative samples demonstrated more activated immune-related pathways and better response to most anticancer agents.

**Discussion:**

Our study revealed a novel risk model and potential feasible prognostic factors for breast cancer and might provide new perspectives for individual breast cancer treatment.

## 1 Introduction

Breast cancer (BRCA) has become the most commonly diagnosed cancer in women. The diagnosis and treatment of BRCA are largely based on the traditional classification of the disease based on the expression of three key receptors: the estrogen receptor (ER), progesterone receptor (PR), and human epidermal growth factor receptor 2 (HER2) (1). Although overall survival has improved in recent decades, a large number of patients still suffer from a poor response to treatments, which is largely attributed to the heterogeneity of the tumor microenvironment (TME) in BRCA (2).

Hypoxia is a canonical characteristic of an adverse microenvironment that weakens most cell functions. Tumor cells are often able to adapt themselves to hypoxia by acquiring malignant abilities, leading to the progression of disease and therapeutic resistance (3). It was reported that exposure to chronic hypoxia could promote cancer cell survival upon reoxygenation by acquiring a reactive oxygen species (ROS)-resistant phenotype and increasing the probability of successful metastasis (4). Several methods have been used for *in vivo* oxygen content measurement and hypoxia observation in BRCA directly or indirectly (5–7), with emerging O_2_ imaging techniques applied clinically. However, the lack of convenience and productivity of these measurements limited the in-depth application of hypoxia research on BRCA to some degree (8).

In this study, patient data from The Cancer Genome Atlas (TCGA) BRCA database were divided into immune-activated and immune-suppressed populations, and the immune-suppressed group was distinguished into hypoxic and non-hypoxic clusters. By defining six key hypoxia-related genes, a hypoxia-related risk model of BRCA was ultimately constructed, and a risk score calculating formula was established. Patients with different ER/PR and HER2 status showed distinct characteristics in risk score, drug sensitivity, immune pathways, and TME composition. Margin status, American Joint Committee on Cancer (AJCC) stage, hypoxic status, ER/PR status, nuclear factor interleukin-3 regulated (NFIL3), serpin family E member 1 (SERPINE1), epidermal growth factor receptor (EGFR), and risk score were noted as independent prognostic indicators for BRCA patients, which was confirmed *via* immunohistochemical staining with a BRCA microarray. This study might provide novel prognostic factors for BRCA research and help in further elucidating the role of hypoxia in different BRCA subtypes.

## 2 Materials and methods

### 2.1 Dataset and preprocessing

TCGA BRCA dataset was selected for the analyses, and the raw data including clinical information, gene expression, and survival data for BRCA were downloaded from the University of California–Santa Cruz (UCSC) Xena (https://xenabrowser.net/datapages/), as detailed in the attachment.

We used the R language (version 4.0.5) idmap1 package (9) to re-annotate the expression data, followed by removal of normal samples (retaining only the tumor samples) and extraction of mRNA expression data according to the HUGO Gene Nomenclature Committee (HGNC) database, which provided the human genome gene information so that a new expression profile for subsequent analysis was constructed. After processing, we obtained the expression levels of 18,012 mRNAs in 1,097 BRCA tumor samples.

### 2.2 Distinguishing between immune-activated and immune-suppressed populations

Single-sample gene set enrichment analysis (ssGSEA) was performed to process TCGA-BRCA data using the R language GSVA package<1> based on 29 immune-related gene sets; the enrichment score of each sample was calculated to perform sparse hierarchical clustering to divide the samples into two populations. R language estimate package (10) was used to calculate the immunopurity of each sample. Differences in key immune markers between the two clusters were statistically analyzed using a t-test.

### 2.3 Distinguishing between hypoxic and non-hypoxic populations

Hierarchical clustering was used to divide the immuno-suppressed samples into hypoxic and non-hypoxic clusters based on the expression of 200 hypoxic marker genes. Notably, among the 200 hypoxia marker genes, nine genes were not annotated because of defective information, and so only the remaining 191 hypoxia marker genes were used in subsequent analyses. Differential analyses of these 191 hypoxia-related genes in the two populations were performed using the R language limma package (11), with the threshold set to |logFC| > 1 and adj-p value<0.01 to obtain the hypoxia-related differentially expressed genes (DEGs).

### 2.4 Analysis of tumor immune microenvironment

CIBERSORT is an R package of deconvolution of the expression matrix of human immune cell subtypes based on the principle of linear support vector regression (linear support vector regression). It is a web version tool using a deconvolution algorithm to estimate the composition and abundance of immune cells out of the mixed cells based on transcriptome data. The referenced gene set including expression signatures is LM22 containing 22 immune cell subtypes. In this study, CIBERSORTx (12) was used to analyze the immune cell infiltration of the expression matrix to obtain the proportion of 22 subtypes of immune cells in BRCA tissues in different groups.

### 2.5 Analysis of tumor drug sensitivity/drug efficacy

The half-maximal inhibitory concentration (IC50) or half-inhibitory rate of a drug is the drug concentration that could kill half of a cancer cell line. It is a very important parameter for measuring the ability of a drug to induce apoptosis; specifically, a drug with a lower IC50 value is better able to induce apoptosis in certain cells, which are thus considered to weakly tolerate the drug. In this study, the R language oncoPredict package (13) was used to predict the drug sensitivity of BRCA tumor samples using the cancer drug sensitivity genomics database (GDSC2) database (which includes 453 drugs, 988 cell lines, and over 380,000 IC50 values) as the training set. The drugs with an average IC50< 1 in all samples were considered specific drugs for BRCA treatment; the sensitivity of these drugs was statistically tested in different groups to explore the different responses of samples with different risk levels to anticancer drugs.

### 2.6 Risk model construction

A risk model was constructed based on the survival data of tumor samples in the database. All samples with a survival status of 0 and survival time<30 days in all samples were regarded as follow-up failures and were removed from overall survival data; the remaining 1,050 samples were used to build the hypoxia-related risk model with the following construction strategy.

A total of 735 samples were randomly selected each time as the training set, in which the key genes related to hypoxia were analyzed *via* multifactor regression analysis in this set, and the genes demonstrating statistical significance were recorded. The above steps were repeated 1,000 times to obtain 1,000 results from the multivariate regression analysis of the randomly selected 70% of the samples. Across the 1,000 repeats, the genes that were determined to be statistically significant in the multivariate regression analyses were counted, and those genes that appeared more than 100 times were regarded as high-frequency risk genes and used to construct the hypoxia risk model.

### 2.7 Analysis of differences and enrichment analysis between subgroups with different ER/PR and HER2 status

Samples containing ER/PR and HER2 grouping information were collected, and the R language limma package was used to perform differential analysis among samples with different ER/PR and HER2 status. For comparing ER/PR+ *vs.* ER/PR−, the threshold was set as |logFC| > 2 and adj-p value ≤0.01. For comparing HER2+ *vs.* HER2−, the screening threshold for differential analysis was set to |logFC| > 0.585 and adj-p value ≤0.01 to obtain an appropriate number of genes for subsequent enrichment analysis. The difference analysis results were visualized in volcano plots and cluster heatmaps.

Gene Ontology (GO) function and Kyoto Encyclopedia of Genes and Genomes (KEGG) pathway enrichment analysis were performed on the DEGs between ER/PR+ and ER/PR− groups based on the R language cluster profiler package (14) to explore the molecular biological functions that might be different between ER/PR+ and ER/PR− groups. At the same time, the clusterProfiler package was used to perform gene set enrichment analysis (GSEA) based on the above differential expression analysis results in order to obtain the activated/inhibited pathways between different groups and explain the potential and possible mechanisms of the disease. Data in HER2+ and HER2− groups were processed in the same way.

### 2.8 Clinical correlation analysis

The R language survminer package (15) was used to calculate the optimal cutoff values for the genes included in the risk model and the risk score to overall survival (OS) data based on gene expression and sample survival data. Multivariate regression analysis of overall survival was conducted according to different clinically relevant phenotypes, followed by 3- and 5-year calibration curve analysis according to different clinical characteristic models.

### 2.9 Clinical sample evidence

The breast cancer tissue microarrays (HBreD140Su06) were purchased from Shanghai Xinchao Biotechnology Co., Ltd. (Shanghai, China). The chip contains 140 breast cancer tissue sites, and seven sites were excluded because of detected flaws. SERPINE1 and FOS expression was measured *via* immunohistochemical staining. The use of tissue chips was approved by the Clinical Research Ethics Committee of Shanghai Xinchao Biotechnology Co., Ltd. (approval number: YBM-05-02).

### 2.10 Statistical description

All data processing and analyses in this analysis were completed in R language (version 4.1.0). The original data were downloaded from the UCSC Xena database and were preprocessed using log2 (count + 1) normalization. The differential gene expression analysis was carried out using the R language limma package. Differences between two groups of continuous variables were estimated using a t-test; hierarchical clustering was used for sample grouping, and Pearson’s correlation was used to calculate the correlation between gene expression and immune cell composition in the TME of BRCA. Survival analysis was performed using the R language survminer package to calculate the optimal cutoffs, followed by the R language survival package on the grouping results; Kaplan–Meier (K-M) curves were plotted to illustrate the survival differences, which were then assessed using the log-rank test. Multivariate regression was used for hypoxia risk model construction and clinical prognosis analysis. In this article, P value calculation was described in corresponding figure legend. P < 0.05 was considered to be significant. *P < 0.05, **P < 0.01, ***P < 0.001, ****P < 0.0001.

## 3 Results

### 3.1 Classification of immune-activated and immune-suppressed populations

**Figure 1:**
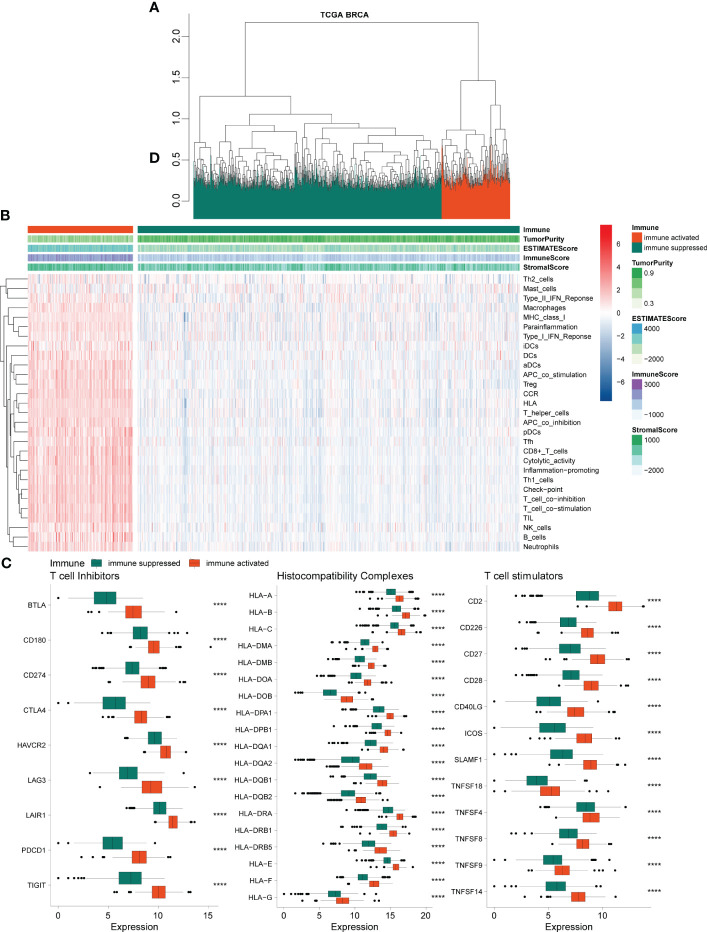
Chromatographic clustering analysis of TCGA BRCA data. **(A)** Chromatographic clustering analysis of samples based on the immune-related scores from ssGSEA of the 29 immune-related gene sets. **(B)** Heatmap of the ssGSEA and immune-related score of 29 immune-related gene sets. **(C)** Analysis of the expression levels of T-cell inhibitors, major histocompatibility complex, and T-cell stimulators. TCGA, The Cancer Genome Atlas; BRCA, breast cancer; ssGSEA, single-sample gene set enrichment analysis.

Based on 29 immune-related gene sets, we calculated the enrichment score of each sample in TCGA-BRCA data using ssGSEA and hierarchical clustering and obtained two groups, referred to as immune-activated (cinnabar red) and immune-suppressed (emerald green) populations (**Figure 1A**). Patients in the immune-suppressed group had lower immune scores and stromal scores, higher tumor purity (**Figure 1B**), and lower immune-related gene expression levels (**Figure 1C**) compared with those in the immune-activated group. The expression level of T-cell inhibitors, major histocompatibility complex, and T-cell stimulators was also significantly reduced in the immune-suppressed cluster (**Figure 1C**).

### 3.2 Further differentiation between hypoxic and non-hypoxic populations in immune-suppressed population

**Figure 2:**
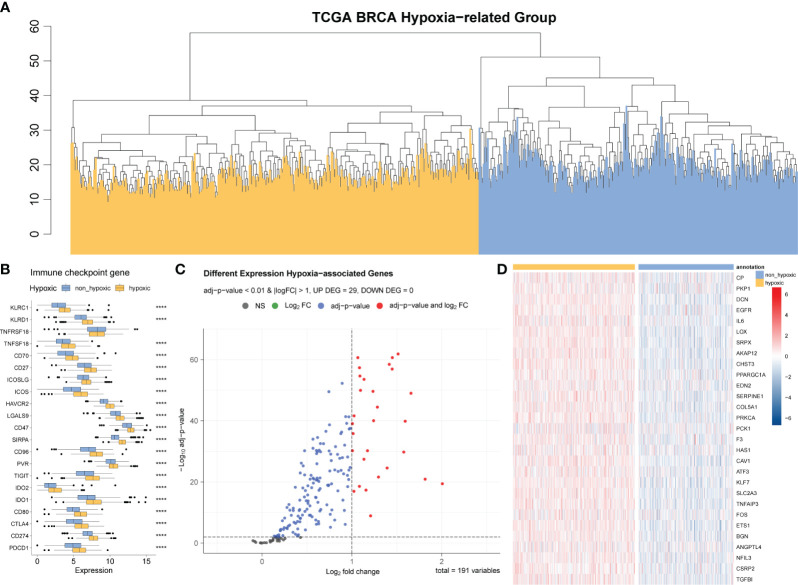
Differential analysis of hypoxic and non-hypoxic populations. **(A)** Hierarchical clustering analysis based on 191 hypoxia-related genes; the samples are divided into two groups: hypoxic and non-hypoxic. **(B)** Differential expression of relevant immune checkpoints in hypoxic and non-hypoxic groups. **(C)** Volcano plot of the differences in the expression of 191 hypoxia-related genes between the hypoxia and non-hypoxia groups. **(D)** Cluster heatmap of the 29 hypoxia-related genes in the two groups of hypoxia and non-hypoxia.

We identified 200 hypoxia-related genes, defined as the HALLMARK_HYPOXIA gene set (**Supplementary Table 1**) and genes upregulated in response to low oxygen levels (hypoxia) from the GSEA website; ultimately, the expression data of 191 hypoxia-related genes were obtained in our expression matrix according to the annotation method described above. Based on these expression data, we further divided the samples in the immuno-suppressed group (described in Section 3.1) into the hypoxic group (goose yellow) and the non-hypoxic group (black blue) using hierarchical clustering method (**Figure 2A**). The volcano plot demonstrates the 191 hypoxia-related genes differentially expressed between the hypoxia and non-hypoxia groups (**Figure 2C**), and the cluster heatmap demonstrates the expression of 29 hypoxia-related genes in the two groups of hypoxia and non-hypoxia (**Figure 2D**). It was indicated that in the hypoxic group, almost all the hypoxia-related genes were significantly upregulated (**Figure 2D**), and most of the immune checkpoint genes were highly expressed (**Figure 2B**).

According to the differential gene analysis and the screening thresholds described in the Materials and Methods section, we obtained 29 genes that were significantly differentially expressed between the hypoxic group and the non-hypoxic group; we then used these significantly hypoxia-related genes to construct the hypoxia-related risk model.

### 3.3 Drug sensitivity analysis and tumor immune microenvironment analysis

**Figure 3:**
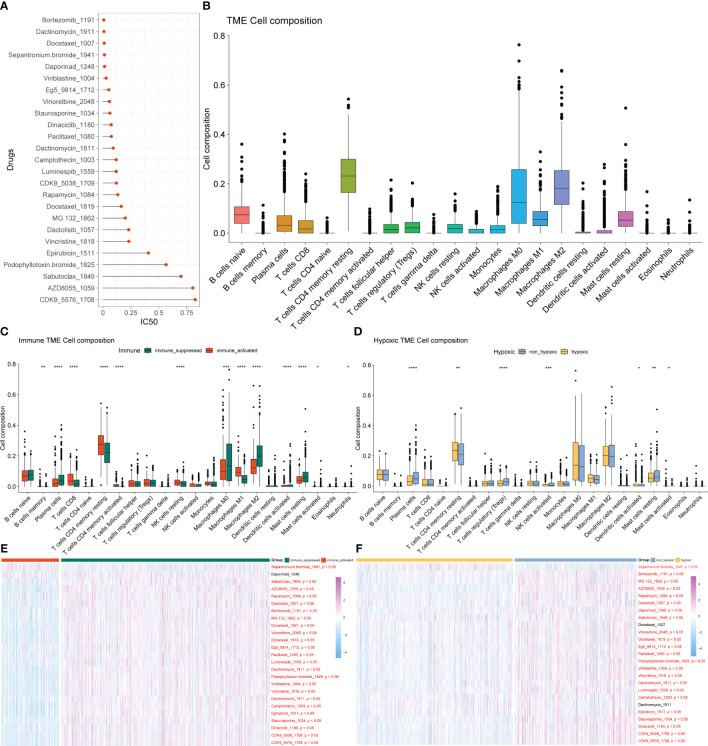
Drug sensitivity analysis and tumor immune microenvironment analysis. **(A)** Drugs with an average IC50< 1 in all samples. **(B)** Composition ratio of 22 immune cells in the TME of patients with BRCA. **(C)** Analysis of immune cell composition differences in immune-activated and immune-suppressed groups. **(D)** Analysis of immune cell composition differences between the hypoxia and non-hypoxia groups. **(E)** Heatmap of different drug responses in immune-activated and immuno-suppressed groups. **(F)** Heatmap of different drug responses in the hypoxia and non-hypoxia groups. TME, tumor microenvironment; BRCA, breast cancer.

As described in the Materials and Methods section, we analyzed the sensitivity of 198 drugs in BRCA, employing the IC50 value as an indicator of drug sensitivity or efficacy, as it is commonly used to assess the therapeutic response to drugs: smaller IC50 value corresponds to better potential therapeutic effects of the drug on BRCA. Those drugs with an average IC50< 1 in all samples are almost exclusively used in the first-line clinical treatment of BRCA (**Figure 3A**).

BRCA is not only an isolated variant cell population but also a microenvironment system composed of cancer cells, immune cells, fibroblasts, fat cells, and endothelial cells, which were gathered inside and around tumors. There are inextricable links and comprehensive cross-talk between immune cells and cancer cells, which are crucial to the biological behavior and therapeutic response of BRCA. The diversity of immune cells makes the analysis of the TME or immune infiltration essential for determining the proportion of immune cells in tumor tissue. We conducted the analysis of the composition ratio of 22 immune cells in the TME of patients with BRCA (**Figure 3B**) and identified significant differences in immune cell infiltration and drug response between the immune-activated and immune-suppressed groups (**Figure 3C**) and between the hypoxia and non-hypoxia groups (**Figure 3D**). The response to most drugs was better in the immune-activated group than in the immuno-suppressed group, indicating the potential for a better therapeutic effect of these drugs in the former (**Figure 3E**). Among the immuno-suppressed samples, the hypoxic group exhibited a better response to cancer treatment drugs than the non-hypoxic group did (**Figure 3F**).

### 3.4 Construction of hypoxia-related scoring system

**Figure 4:**
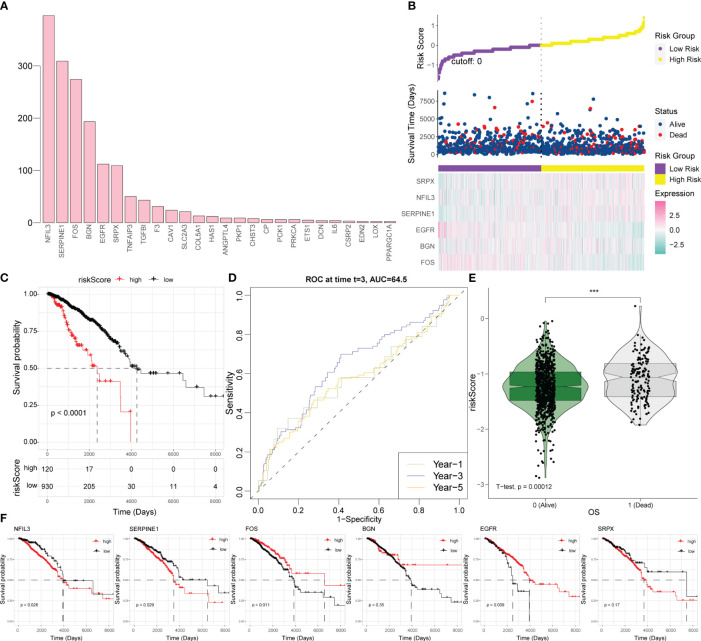
Establishment of a hypoxia-related scoring system. **(A)** Statistical analysis of the significant results of the multivariate regression analysis in 70% of the samples randomly collected 1,000 times. **(B)** Risk triad of the prediction model. **(C)** Survival analysis based on the hypoxia risk score. **(D)** Time-ROC curve analysis based on the hypoxia risk score. **(E)** Survival status test based on the hypoxia risk score. **(F)** Survival analysis results for six key hypoxic model genes in BRCA. ROC, receiver operating characteristic; BRCA, breast cancer.


Based on the 29 significantly differentially expressed hypoxia-related genes described in Section 3.2, we randomly selected 70% of the BRCA tumor samples 1,000 times to conduct multivariate regression analysis and counted the significant genes in each regression analysis result (**Figure 4A**). Genes with frequency exceeded 100, which were NFIL3, SERPINE1, FOS, biglycan (BGN), EGFR, and sushi-repeat-containing protein, X-linked (SRPX), were adopted as high-frequency genes and potential key factors in the hypoxia model to calculate hypoxia-related scores. The resulting risk score was calculated as follows:

$$\begin{array}{l} riskScore\;=\;-0.\;1637688\;*\;FOS\;+\;0.\;1551399\;*\;SERPINE1\;+\;0.\;1672584\;*\;NFIL3\\ -0.\;1583654\;*\;BGN-0.\;2149130\;*\;EGFR\;+\;0.2194696\;*\;SRPX. \end{array}$$

According to this model, the expression levels of SRPX, NFIL3, and SERPINE1 increase along with the increase of survival risk, indicating that they are positive high-risk factors; the expression levels of FOS, BGN, and EGFR decrease with increasing survival risk, representing negative high-risk factors (**Figure 4B**). For BRCA patients, a higher hypoxia score was associated with worse survival (**Figure 4C**). For survival prediction verification, the hypoxia-related score demonstrated the highest predictive value for 3-year survival in patients with BRCA (**Figure 4D**). Patients who died of the disease had a statistically significantly greater hypoxia score than patients who survived (**Figure 4E**); consistent with the results of the hypoxia scoring model, SRPX, NFIL3, and SERPINE1 were risk factors for BRCA prognosis and survival, while FOS, BGN, and EGFR were protective factors for BRCA prognosis and survival (**Figure 4F**).

### 3.5 Relationships between key hypoxia factors and tumor immune microenvironment of BRCA

**Figure 5:**
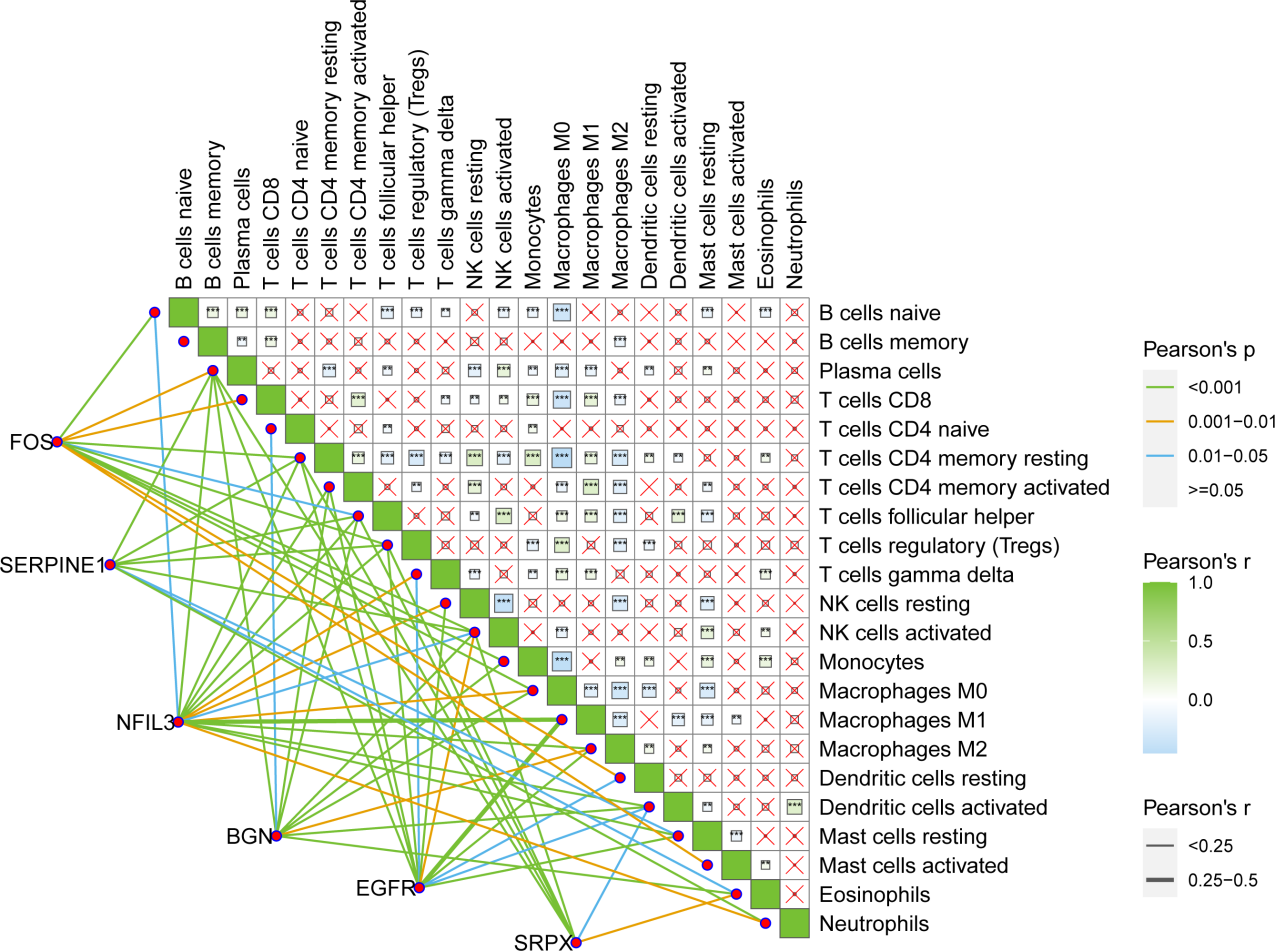
Pearson’s interaction between six key hypoxia factors and 22 immune cell components in the TME of BRCA patients and the interactions between immune cells. TME, tumor microenvironment; BRCA, breast cancer.

Pearson’s correlation analysis was conducted between six key hypoxic factors and 22 immune cell components in the TME and the interaction between the immune cells. The results showed that the six genes were correlated with the given immune cells (**Figure 5** **).**

### 3.6 Analysis of clinical correlation

**Figure 6:**
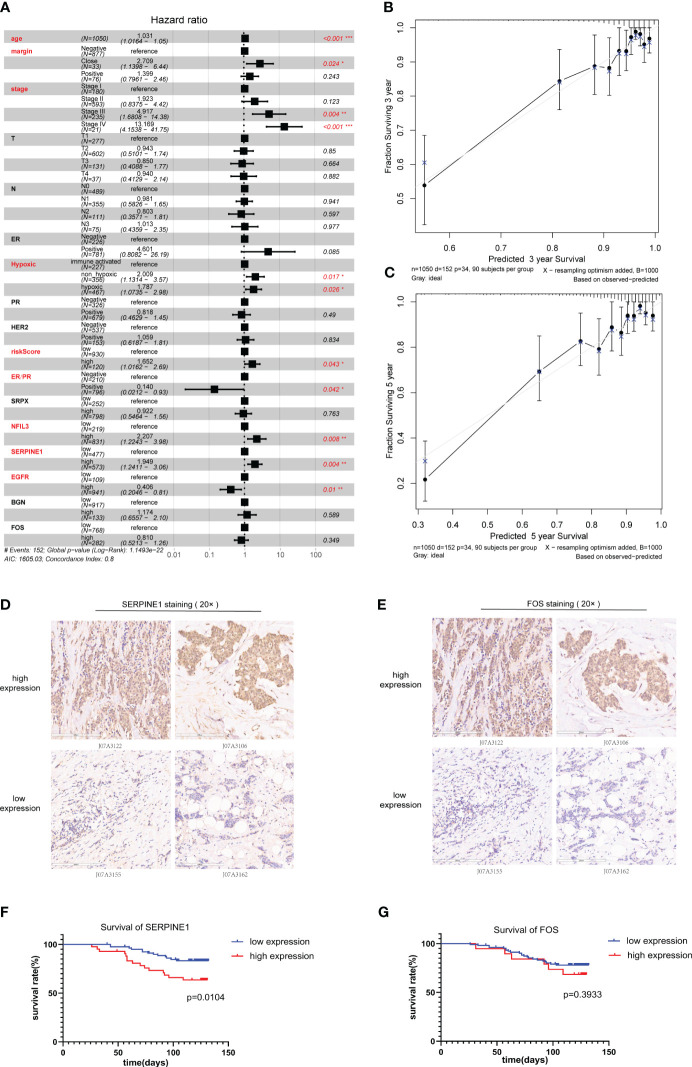
Clinical prognostic analysis. **(A)**. Multivariate regression prognostic analysis of clinical characteristics related to BRCA. **(B)**.Calibration curve for 3-year survival. **(C)**. Calibration curve for 5-year survival. **(D, E)** Immunohistochemical staining for SERPINE1 **(D)** and FOS **(E)** expression in samples of breast cancer tissue microarrays. **(F, G)** Kaplan–Meier analysis of the correlation between SERPINE1 **(F)** and FOS **(G)** expression and the overall survival rate of patients in the BRCA cohort according to the microarray data. BRCA, breast cancer.

The optimal cutoff values of the genes included in the risk model and the risk score to OS data were calculated based on gene expression and sample survival data. Samples that exceeded the optimal cutoff were defined as the high-expression or high-risk score group, and samples that did not exceed the optimal cutoff value were defined as the low-expression or low-risk score group. Detailed information on each sample is shown in **Supplementary Table 2**. Multivariate regression analysis for OS was conducted based on different clinically relevant phenotypes grouped by gender, age, margin status, AJCC T/N/M stage, HER2 status, ER/PR status, immune status, and hypoxic status. The results showed that margin status, AJCC stage, hypoxia status, ER/PR status, NFIL3, SERPINE1, EGFR, and risk score could be used as independent prognostic indicators for BRCA patients (**Figure 6A**). Among them, a close margin status, AJCC stage III/IV, immune suppressed status (non-hypoxic + hypoxic, non-hypoxic > hypoxic), high hypoxic risk score, negative ER/PR, high NFIL3 level, high SERPINE1 level, and low EGFR level were found to be prognostic factors for BRCA. According to the results of multivariate regression analysis based on clinical characteristics, 3- and 5-year calibration curves of the clinical characteristic model were plotted to visualize the statistical significance of the clinical characteristics on the prognosis of patients with BRCA (**Figures 6B, C**). Among the clinical samples, the levels of SERPINE1 and FOS were measured as representative markers for verification. Breast cancer tissue microarrays (No. HBreD140Su06) harboring clinicopathological characteristics, including tumor size, histological grade, lymph node stage, and metastasis, and survival information were used to investigate the correlation between SERPINE1 or FOS expression and clinicopathological characteristics in 123 cancer patients (samples with incomplete information were excluded) (**Supplementary Table 3**). The expression of SERPINE1 and FOS was determined by immunohistochemical analysis (**Figures 6D, E**; **Supplementary Tables 4**, **5**). As shown in the K-M survival curves, univariate analysis showed a significant correlation between SERPINE1 expression and the overall survival rate, which indicated that low SERPINE1 levels were a protective prognostic factor for BRCA patients (**Figure 6F**; **Table 1**), whereas low FOS expression showed a non-significant correlation with better survival (**Figure 6G**; **Table 2**), which is consistent with our prediction in **Figure 6A**.

**Table 1:** Analysis of SERPINE1 expression and clinical characteristics.

Characteristics	All cases (n = 123)	SERPINE1 expression		χ^2^	p-Value
		Low (n = 81)	High (n = 42)		
Age (years)					
≤55	68	45	23	0.007	0.933
>55	55	36	19		
Pathological grade					
1–2	86	60	26	1.947	0.163
3–4	37	21	16		
T					
T1	55	36	19	0.007	0.933
T2	68	45	23		
N					
N0	66	43	23	0.031	0.86
N1–3	57	38	19		
Recurrence					
No	92	64	28	2.236	0.135
Yes	31	17	14		
Clinical grade					
1–2	83	53	30	0.453	0.501
3	40	28	12		
Survival status					
Alive	95	68	27	6.083	0.014
Dead	28	13	15		

**Table 2:** Analysis of FOS expression and clinical characteristics.

Characteristics	All cases (n = 123)	FOS expression		χ^2^	p-Value
		Low (n = 104)	High (n = 20)		
Age (years)					
≤55	68	56	12	0.563	0.453
>55	55	48	7		
Pathological grade					
1–2	86	77	9	5.433	0.02
3–4	37	27	10		
T					
T1	53	41	12	3.691	0.555
T2	70	63	7		
N					
N0	66	54	12	0.815	0.367
N1–3	67	50	7		
Recurrence					
No	92	80	12	1.615	0.204
Yes	31	24	7		
Clinical grade					
1–2	83	70	13	0.009	0.924
3	40	34	6		
Survival status					
Alive	95	82	13	0.993	0.319
Dead	28	22	6		

### 3.7 Comparison between the ER/PR+ and ER/PR− groups

**Figure 7:**
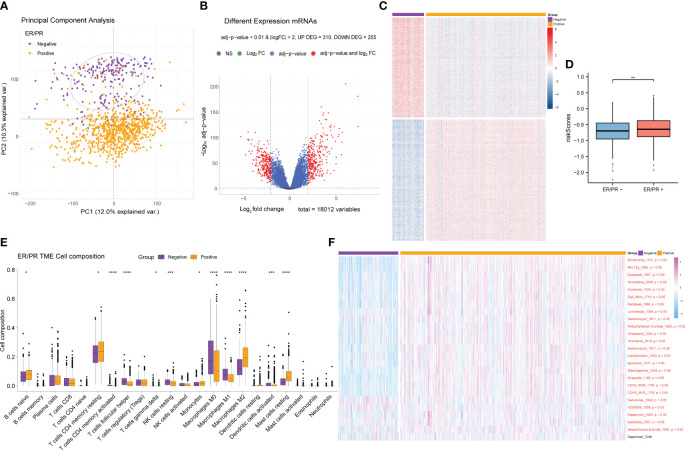
Differential gene expression analysis of the ER/PR+ and ER/PR− groups and comparative analysis results of tumor microenvironment and drug response. **(A)** PCA of the gene expression of the ER/PR+ and ER/PR− groups. **(B)** Volcano plot of the ER/PR grouping difference analysis. **(C)** Cluster heatmap of the ER/PR grouping difference analysis. **(D)** Risk score comparison between the two groups. **(E)** Differential analysis of tumor immune cell infiltration in the two groups. **(F)** Difference analysis of drug treatment efficacy. ER, estrogen receptor; PR, progesterone receptor; PCA, principal component analysis.

ER and PR are important receptors in the BRCA classification system; therefore, samples containing ER/PR grouping information were collected, and differential analysis of the ER/PR+ and ER/PR− groups was performed using the R language limma package (**Supplementary Table 6**). Principal component analysis (PCA) revealed that the genes in the two groups exhibited distinct expression patterns (**Figure 7A**), which were visualized *via* volcano plots (**Figure 7B**) and cluster heatmaps (**Figure 7C**; **Supplementary Table 7**). According to our risk formula, ER/PR+ patients seemed to have higher hypoxia-related risk scores (**Figure 7D**). There were also statistically significant differences in tumor immune microenvironment (**Figure 7E**) and response to drug treatment (**Figure 7F**) between the two groups. These findings revealed that ER/PR− patients might respond better to most tumor treatment agents.

### 3.8 Enrichment analysis of differentially expressed genes between ER/PR+ and ER/PR−

**Figure 8:**
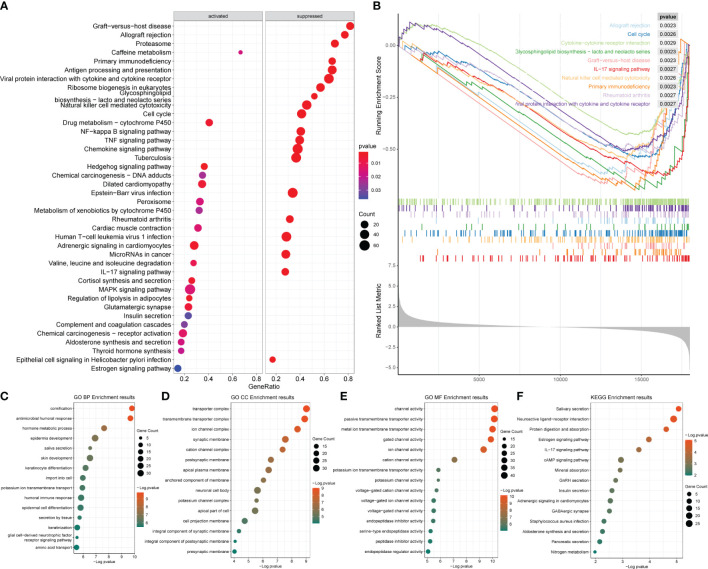
Enrichment analysis results of the differentially expressed genes between ER/PR+ *vs.* ER/PR− breast cancer. **(A)** Bubble diagram of the GSEA results for the top 20 genes. **(B)** GSEA results with top 10 NESs. **(C–E)** GO BP/CC/MF enrichment analysis. **(F)** KEGG enrichment analysis. ER, estrogen receptor; PR, progesterone receptor; GSEA, gene set enrichment analysis; NESs, normalized enrichment scores; GO, Gene Ontology; BP, biological process; CC, cellular component; MF, molecular function; KEGG, Kyoto Encyclopedia of Genes and Genomes.

After obtaining the differentially expressed genes between the ER/PR+ and ER/PR− groups, GO function and KEGG pathway enrichment analysis were performed based on the R language clusterProfiler package to explore the molecular biological functions of the two groups. Significant functional differences in immune and metabolism-related molecular functions and pathways were revealed under different ER/PR status (**Figure 8A**). ClusterProfiler package was also used to perform GSEA to determine differences in the pathways activated/inhibited between the two groups to explain the potential underlying mechanisms of the disease. The ER/PR-positive group obtained activated metabolism-related pathways and inhibited immune-related pathways (**Figure 8B**); compared to those of the opposite group, the metabolism-related pathways in the tumor tissues of the ER/PR-positive patients were significantly activated, while the immune-related pathways in the tumor tissues of ER/PR-negative patients were significantly activated. GO analyses on the biological process (BP), cellular component (CC), and molecular function (MF) of the differentially expressed genes between the ER/PR-positive and ER/PR-negative groups are demonstrated in **Figures 8C–E**, and KEGG analysis is demonstrated in **Figure 8F**.

### 3.9 Comparison between the HER2-positive and HER2-negative groups

**Figure 9:**
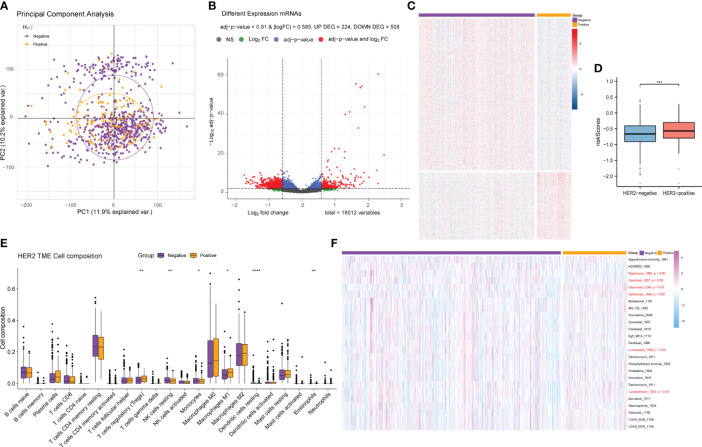
Differential gene expression analysis and comparative analysis of the tumor microenvironment and drug treatment efficacy between the HER2-positive and HER2-negative patients. **(A)** PCA of the gene expression of HER2-positive and HER2-negative samples. **(B)** Volcano plot of the difference analysis results of HER2-positive and HER2-negative samples. **(C)** Cluster heatmap of the difference analysis results for the two groups. **(D)** Risk score comparison between the HER2+ and HER2− groups. **(E)** Differences in tumor immune microenvironment cell composition between the HER2-positive and HER2-negative groups. **(F)** Differences in drug response between the two groups. PCA, principal component analysis.

HER2 is another vital marker in BRCA classification, and its status determines the biological behavior, treatment, and prognosis of BRCA. We also collected samples containing HER2 information and performed differential gene analysis between the HER2-positive and HER2-negative groups in the same way as the ER/PR group analysis (**Supplementary Tables 8**, **9**). However, we did not observe significant differences in gene expression between the HER2-positive and HER2-negative samples (**Figures 9A–C**), and there was no significant difference in either the TME or drug response between the two groups (**Figures 9E, F**). However, the HER2+ patients had a higher hypoxia-related risk score (**Figure 9D**).

### 3.10 Enrichment analysis of HER2-positive and HER2-negative differential genes

**Figure 10:**
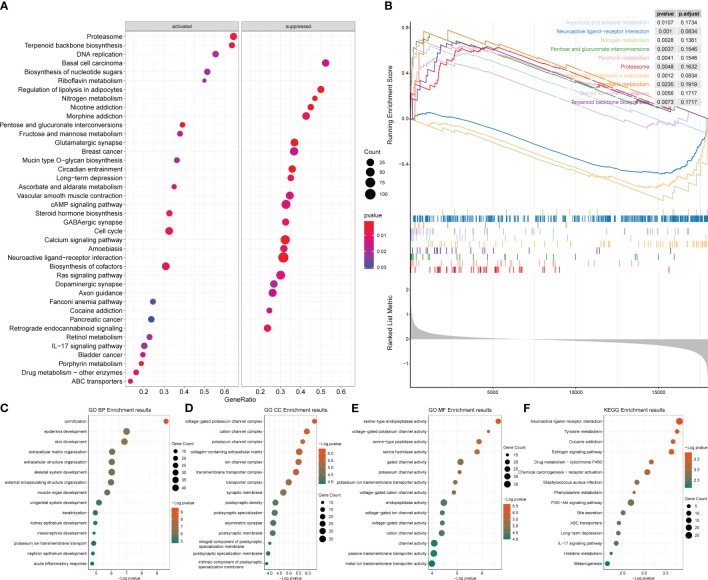
Enrichment analysis results of the differentially expressed genes between HER2-positive and HER2-negative breast cancer. **(A)** Bubble diagram of the top 20 genes identified by GSEA. **(B)** GSEA results of the top 10 NESs. **(C–E)** GO enrichment analysis. **(F)** KEGG enrichment analysis of the two groups. GSEA, gene set enrichment analysis; NESs, normalized enrichment scores; GO, Gene Ontology; KEGG, Kyoto Encyclopedia of Genes and Genomes.

Enrichment analysis was also conducted for the HER2-positive and HER2-negative groups as mentioned in Section 3.9 (**Supplementary Table 4**). The analysis revealed that the main pathways enriched in the differentially expressed genes included metabolic and immune-related pathways (**Figures 10A, B**), while no significant activation preference was identified (**Figures 10C–F**). GO analyses on the BP, CC, and MF of the differentially expressed genes between the HER2-positive and HER2-negative groups are demonstrated in **Figures 10C–E**, and KEGG analysis is demonstrated in **Figure 10F**.

## 4 Discussion

BRCA remains one of the leading causes of cancer death in women (16). However, mortality of the disease remains high due to the development of metastasis and the emergence of drug resistance. Numerous studies have shown that the proliferation and metastasis potential of malignant cells are strongly influenced by the TME. Hypoxia is a canonical feature of a low-oxygen level environment, and it is vital to physiological and pathological mechanisms (17). This condition plays important roles in nearly all aspects of BRCA, including oncogenesis, invasion, metastasis, and drug resistance as results of the process of cancer cells adapting to hypoxic stress. Hypoxia also helps promote tumor cells escape from immune surveillance and even immunotherapy (18). There is evidence that hypoxia in the TME might suppress the expression of immune effector genes in T and natural killer (NK) cells, resulting in immune cell dysfunction and resistance to immunotherapy (19). Clinical studies have demonstrated that hypoxia is associated with immune evasion, leading to therapeutic resistance to immune checkpoint inhibitors in some tumors (20–22). In BRCA, the hypoxic phenotype was considered a prognostic factor for tumor relapse and poor survival regardless of the subtypes and stages of the disease (23). Several other reports have revealed that hypoxia in the TME might induce T-cell exhaustion in mice, and deprivation of hypoxia-inducible factor-1 alpha (HIF-1α) could increase NK cell activity and infiltration in tumor areas (24).

For BRCA treatment, it is widely assumed that tumor hypoxia is related to a negative response to chemo- or radiotherapy and that targeting hypoxia should be included in BRCA therapy (25). HIF-1α was one of the hypoxia markers that was previously investigated in numerous studies, as reports have shown a lower rate of pathological complete response (PCR) with higher HIF-1α expression in BRCA patients (26, 27); however, different strategies, such as targeting key players in metabolic and angiogenic pathways and even bevacizumab in BRCA, have yielded unsatisfactory results (28). Therefore, there is an urgent need for additional potential markers and the identification of links between hypoxia and BRCA treatment.

In this study, after ssGSEA based on 29 immune-related gene sets, the BRCA samples from TCGA database were sparsely stratified and clustered into two populations: immune activation (cinnabar red) and immunosuppression (emerald green). Patients in the immuno-suppressed group had lower immune scores, lower stroma scores, and higher tumor purity, along with significantly lower expression levels of T-cell inhibitors, major histocompatibility complex, and T-cell stimulators. These results illustrated the heterogeneity of immune status in the TME and may be closely related to the treatment response and prognosis of BRCA patients.

In the following analyses, the hypoxia and non-hypoxia groups were further distinguished from the immuno-suppressed population. In the hypoxia group, most of the immune checkpoint genes had higher expression levels, and almost all hypoxia-related genes were significantly upregulated. This suggested that hypoxia might be an important factor influencing the tumor microenvironment and treatment response, especially in pre-existing immunosuppression. Consistent with the findings of previous studies, the immune-suppressed group presented a poorer response to most BRCA-related drugs, and the non-hypoxic group exhibited a worse response than the hypoxic group did. In other words, our model showed that under immune suppression circumstances, a non-hypoxic condition might exacerbate the suppressive immune microenvironment, which provided novel perspectives on the connections between tumor hypoxia and microenvironment.

In this study, by multiple regression analysis, we obtained genes with high frequency and statistical significance and then constructed a hypoxia-associated risk score model. Six genes including NFIL 3, SERPINE1, SRPX, FOS, BGN, and EGFR were defined as key hypoxia-related factors in this model. NFIL3 is a transcriptional repressor. The abnormal expression of NFIL3 was reported to be a poor prognostic factor in various types of tumors (29–31). Furthermore, the level of NFIL3 protein was significantly increased in triple-negative breast cancer (TNBC) and was associated with poorer prognosis (32). SERPINE1 or plasminogen activator inhibitor 1 (PAI-1) has been reported to be involved in metabolic changes, progression, metastasis, and drug resistance in various cancers, including BRCA (33–36). In TNBC, SERPINE1 was noted to mediate obesity-associated tumor radioresistance (37). In informatic analyses, SRPX has been used to construct a model for predicting the prognosis of colorectal cancer (38) and is another hypoxia-related signature for prognosis prediction in head and neck squamous carcinoma (39). In our study, NFIL3, SERPINE1, and SRPX were considered adverse prognostic risk factors for BRCA, which is consistent with the findings of the literature. Among the protective factors in our model, FOS is a proto-oncogene of the activator protein-1 (AP-1) transcription factor subunit (40) and participates in a variety of cellular functions and apoptotic cell death, including regulating the development and progression of BRCA (41, 42), and its overexpression attenuates the malignant phenotypes of BRCA cells according to the lines of evidence from *in silico* and *in vitro* studies (43). The protein encoded by BGN (biglycan) was proven to induce BRCA cell normalization (44) and inhibit the initial outgrowth of brain metastases in BRCA patients (45). EGFR is generally considered to stimulate cell proliferation and promote cancer cell survival (46). However, in both clinical and preclinical settings, the therapeutic efficacy of EGFR-targeted therapies, including monoclonal antibodies for TNBC treatment, has been unsatisfactory (47). Several studies have attempted to explain the mechanisms underlying the unsatisfactory effects of these strategies (48, 49), suggesting the need for additional exploration. After the identification of the six key hypoxia-related factors, we conducted Pearson’s correlation analysis between six key hypoxic factors and 22 immune cell components in the TME and the interaction between the immune cells. Understanding the effects of hypoxia on immune infiltration, especially immunosuppression, would contribute to better exploring the mechanisms of tumor development and therapeutic resistance in breast cancer. This might also provide certain guidance for the development of combined treating strategies against immunosuppression and hypoxia to improve the clinical outcomes of BRCA patients.

Our hypoxia-related risk score model is able to predict the 3-year survival of BRCA effectively, and the dead patients had higher hypoxia scores than the surviving patients. We verified good correlation through both 3-year and 5-year survival curves. Additionally, the calibration curves of 3- and 5-year survival rates were similar in showing relatively high reliability of the model. In particular, the calibration curve for 3-year survival is also consistent with our findings in **Figure 9D** that the hypoxia-related risk score has the highest predictive value for 3-year survival in BRCA. The margin status, AJCC stage, hypoxia status, and ER/PR status can be used as important indicators for the survival outcome of BRCA patients. In the calculation, the C-index is approaching 0.8 (1–0.204), indicating a clinically relevant model with high confidence. These clinical parameters were combined to provide more comprehensive and precise information about the prognosis of BRCA patients. This reliable and accurate predicted prognostic risk scoring system for BRCA as well as the key genes might provide more prognostic markers and some potential targets for BRCA research and treatment.

Moreover, we estimated different signatures of major pathways between ER/PR− and ER/PR+ clusters and found that samples with ER/PR+ and ER/PR− showed statistically significant differences in gene expression, tumor immune microenvironment, and response to drug treatment. There are more activated metabolism-related pathways in ER/PR+ populations along with more inhibited immune-related pathways in ER/PR− populations. The role of ERα in regulating the balance and homeostasis of energetic metabolism was revealed to be linked to its classical nuclear activity (50), upon which our analyses might provide supportive data and the necessity of metabolism regulation in BRCA treatment. We also demonstrated that the immune-related pathways in tumor tissues of ER/PR-negative patients were significantly activated, which is bioinformatically consistent with the acknowledgment that HR-positive BRCA tends to have fewer immune infiltrates and be more immunogenic than HR-negative microenvironment (51–53). In our analyses, samples of ER/PR− might respond better to BRCA-related drugs. These findings reveal the importance of estrogen receptor and progesterone receptor status for breast cancer biological characteristics and the selection of therapeutic strategies.

It is noteworthy that the HER2+ and HER2− samples showed no significant statistical differences in gene expression, tumor immune microenvironment, and response to drugs in our study. This implies that HER2 status might not be the only or key decisive indicator of breast cancer cure sensitivity or medication.

In this study, we found some significant differences in metabolism and immune pathways among groups with different hormone receptor status. Similar associations have been found between HIF-1α and other markers used to identify BRCA classes, including the hormone receptor and HER2 status. In some studies, HIF-1α levels in the tissues of BRCA patients correlated with ERα expression and HER2 positivity (54–56). Interestingly, a greater hypoxia-related risk score was estimated in the ER/PR+ and HER2+ groups. For the first time, we reported the associations between hypoxia signatures and different hormone receptor or HER2 status. Future studies should further investigate hypoxia among patients with different BRCA subtypes.

There are several limitations to our study. First, the raw data were mainly from TCGA database, and additional databases should be involved in future analyses, which should take batch-to-batch variation into consideration. It is worth noting that as a highly aggressive and hypoxia-associated subtype of BRCA, TNBC is not separately or specifically investigated in our current study due to an insufficient number of TNBC cases in the collected samples. It is reported that TNBC demonstrated a more pronounced hypoxic signature than other BRCA subtypes (57) (58); even under normoxia, TNBC cells demonstrate a hypoxia gene signature (59). Targeting HIF-1α and the associated epigenetic machinery is reported to be a promising strategy to reverse the immune effector dysfunction and overcome resistance to PD-1 blockade (60). Our future studies will be devoted to accumulative TNBC sample collection in the research projects and deeply analyze the differences in hypoxia and other characteristics with other BRCA subtypes, which calls for continuous data accumulation and deepening research in the future. We look forward to a better understanding of the characteristics and treatment strategies of TNBC and hypoxia so as to contribute to the research and treatment of BRCA.

In addition, the key factors previously reported in the literature that are consistent with our analyses were mostly investigated *via* a bioinformatics approach, and additionally, more solid evidence is needed to support the results, especially that from more biological experiments and clinical verification of the key factors and the relationship between hypoxia signatures and the TME.

## 5 Conclusions

In summary, we distinguished immune-activated and immune-suppressed populations from TCGA database, the latter of which was further divided into hypoxic and non-hypoxic clusters. The drug response was presented better in the immune-activated group than in the immuno-suppressed group and in the hypoxic group than in the non-hypoxic group. A hypoxia-related risk model of BRCA was constructed with high reliability in this study using six key hypoxia-related genes adopted. ER/PR-negative patients exhibited significantly activated immune-related pathways and improved drug response, while ER/PR-positive patients exhibited increased activation of metabolism-related pathways. ER/PR+ and HER2+ patients might have higher hypoxia-related risk scores. Our study could lead to the identification of novel prognostic biomarkers and perspectives on BRCA treatment, especially immune therapy.

## Data availability statement

The datasets presented in this study can be found in online repositories. The names of the repository/repositories and accession number(s) can be found in the article/**Supplementary Material**.

## Ethics statement

The studies involving humans were approved by Clinical Research Ethics Committee of Shanghai Xinchao Biotechnology Co., Ltd. The studies were conducted in accordance with the local legislation and institutional requirements. The participants provided their written informed consent to participate in this study.

## Author contributions

YL: Data curation, Formal analysis, Funding acquisition, Methodology, Resources, Writing – original draft, Writing – review & editing. HY: Data curation, Formal analysis, Methodology, Resources, Software, Validation, Visualization, Writing – review & editing. XH: Conceptualization, Supervision, Writing – review & editing. YP: Conceptualization, Funding acquisition, Supervision, Writing – review & editing.

## Supplementary material

Supplementary Figure 1 Flowchart and design of the analyses. Firstly, gene expression data from the TCGA breast cancer dataset were re-annotated using R language, and yielded gene expression profiles fo BRCA samples. Then, ssGSEA was performed based on immune-related gene sets, and we divided samples into immune-activated and immune-suppressed populations. According to hypoxia marker genes, the immunosuppressed population were further divided into hypoxic and nonhypoxic populations, and differential analysis was performed to obtain differentially expressed genes associated with hypoxia. The immune cell subtype proportions was analyzed based on the expression matrix and the oncoPredict package was used for tumor drug sensitivity prediction and statistical tests. Subsequently,we constructed a hypoxia-related risk model based on the candidate gene set, and performed a series of clinical relevant analysis. Additionally, differential analysis and enrichment analysis were perfromed between ER/PR+ and ER/PR- populations, as well as Her2+ and Her2- populations.
