# Supplementary figures and images for: Analyses of hypoxia-related risk factors and clinical relevance in breast cancer
Source: Front Oncol. 2024 Mar 4;14:1350426. doi: 10.3389/fonc.2024.1350426 (PMC10946248; doi:10.3389/fonc.2024.1350426)

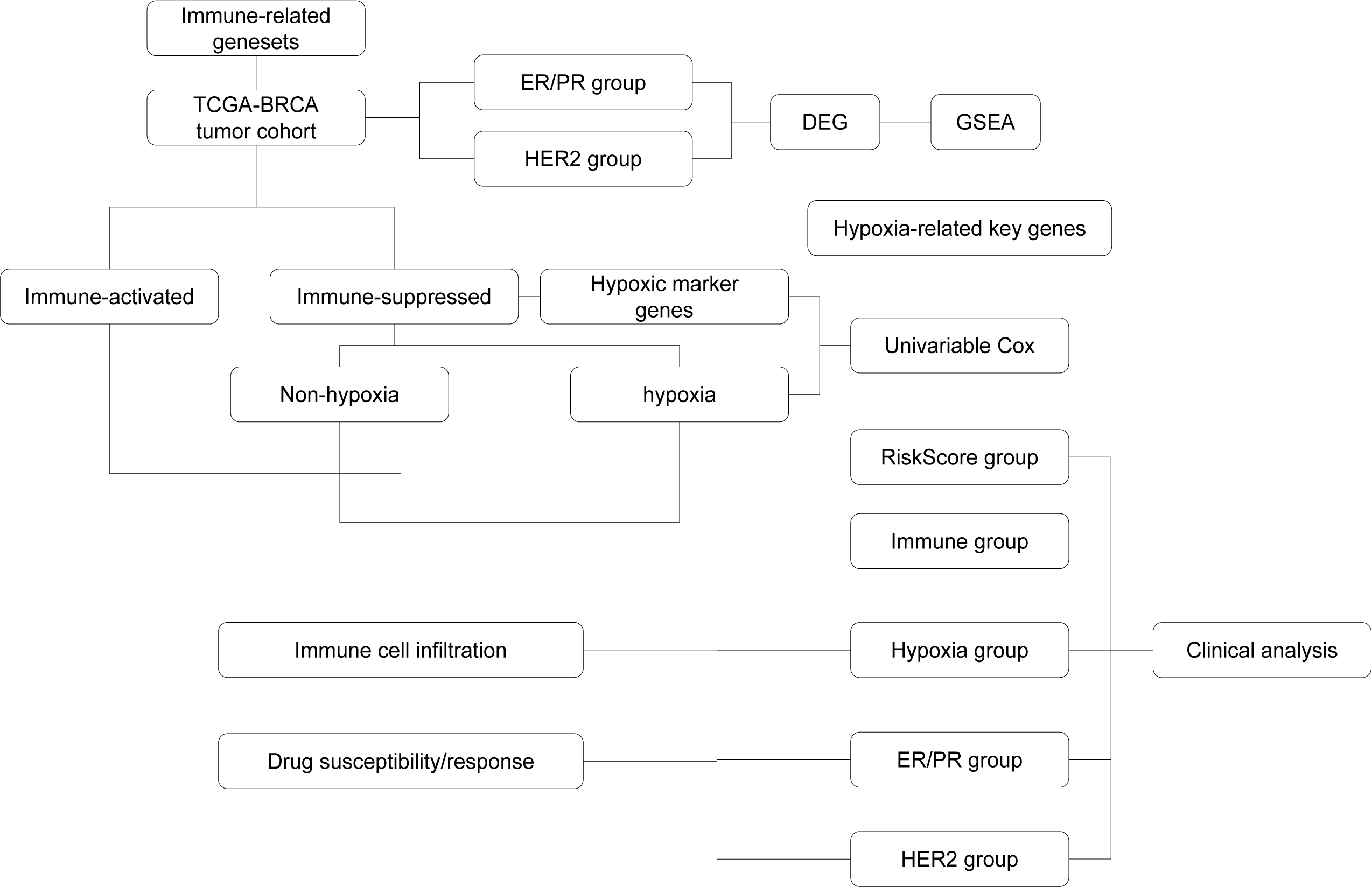

Supplement: Supplementary Figure 1 — Flowchart and design of the analyses. Firstly, gene expression data from the TCGA breast cancer dataset were re-annotated using R language, and yielded gene expression profiles fo BRCA samples. Then, ssGSEA was performed based on immune-related gene sets, and we divided samples into immune-activated and immune-suppressed populations. According to hypoxia marker genes, the immunosuppressed population were further divided into hypoxic and nonhypoxic populations, and differential analysis was performed to obtain differentially expressed genes associated with hypoxia. The immune cell subtype proportions was analyzed based on the expression matrix and the oncoPredict package was used for tumor drug sensitivity prediction and statistical tests. Subsequently,we constructed a hypoxia-related risk model based on the candidate gene set, and performed a series of clinical relevant analysis. Additionally, differential analysis and enrichment analysis were perfromed between ER/PR+ and ER/PR- populations, as well as Her2+ and Her2- populations. [file Image_1.tif]
